# The round trip model for severe herpes zoster caused by live attenuated varicella vaccine virus

**DOI:** 10.1002/jmv.25664

**Published:** 2020-01-17

**Authors:** Charles Grose, Lynn W. Enquist

**Affiliations:** ^1^ Virology Laboratory, Children's Hospital, University of Iowa Iowa Iowa; ^2^ Department of Molecular Biology Princeton University Princeton New Jersey

## Abstract

- Varicella vaccine is a live attenuated varicella‐zoster virus.
- Varicella vaccine can enter latency and later reactivate as herpes zoster.
- Pseudorabies virus is another herpesvirus closely related to varicella‐zoster virus.
- The round trip model for pseudorabies virus explains pathogenesis of herpes zoster from vaccine virus.

The neurovirulence of the live attenuated varicella vaccine virus is a subject of increasing interest.^1^ Since there is no ideal animal model for varicella‐zoster virus (VZV) infection and neuropathogenesis, we have turned to animal models for the closely related pseudorabies virus (PRV).^2^ PRV is the alpha herpes virus of swine; the first live attenuated PRV vaccine (Bartha strain) was produced in 1961 in Hungary, a decade before the live attenuated VZV vaccine (Oka strain) was produced in Japan. Neurologic disease caused by PRV often resembles neurologic disease caused by VZV.^3^ Unlike VZV, PRV replicates in other mammals, such as the dog and rodents. When PRV infects these animals, PRV causes a disease called “mad itch,” a virulent pruritis. Depending on the site of PRV entry into the scarified skin of the mouse model, within 2 days mice will unceasingly scratch the skin adjacent to the site of infection, which leads to a circumscribed lesion precisely defining the dermatome of the innervating dorsal root ganglion (DRG).^4^ Infection of the DRG is necessary for the occurrence of mad itch.

In the current report from the Journal of Medical Virology, the authors found one case of herpes zoster caused by varicella vaccine virus in a child immunized with varicella vaccine.^5^ Other recent reports have described immunized children who developed severe herpes zoster with an extensive dermatomal rash.^6^, ^7^ The most widely held hypothesis is that herpes zoster is the reactivation of virus previously found in the vesicular rash of varicella, that has traveled retrograde in the sensory neurons to the soma in the DRG to establish a latent state.^8^ When virus reactivates in the DRG, the virus travels in the anterograde direction to the dermatome where the vesicles were originally located. In fact, the human dermatomal map was created over a century ago based on correlations between locations of damaged DRGs and herpes zoster.^9^ Based on the same skin vesicle/DRG model, zoster that occurs in an immunized child should be confined to a small area of skin representing the site of the original vaccine injection (often in the thigh), since most injections of vaccine do not lead to a rash around the injection site. However, the dermatomal rash from vaccine‐related herpes zoster can be as extensive as herpes zoster after wild‐type varicella (chickenpox).^6^ Herein we propose the “round trip” model of PRV infection of peripheral nervous system ganglia as an explanation for severe herpes zoster caused by varicella vaccine virus.

The invasion of peripheral ganglia after PRV infection has been studied in the murine salivary gland model.^2^ In this report, the salivary gland is infected with a recombinant PRV expressing a fluorescent capsid protein that assembles into traceable infectious particles. The salivary gland and its adjacent submandibular ganglia are removed on bloc. Fluorescent virus particles are imaged as they are transported via the axons to the submandibular ganglia. Replication initially occurs in a few neurons in the ganglia and progeny virus particles are transported in the anterograde direction back to the salivary gland, where they infect more glandular cells. In turn, new progeny virus particles are transported in the retrograde direction to the ganglia (a round trip). By 48 hours after PRV infection, all neurons in the ganglia are infected. Thus, the neuronal round trips of PRV from the infected salivary gland amplify the infection in the neurons in the ganglia.^2^

The hypothesis of this commentary is that a PRV model for a severe dermatomal disease called mad itch explains severe dermatomal herpes zoster caused by the varicella vaccine virus. After injection of varicella vaccine, usually in the thigh, virus replicates locally and then infects sensory axons, before traveling in the retrograde direction to the lumbar DRG. (Note that 5 of the 6 cases of herpes zoster described in References 5, 6, and 7 were in the leg.) The virus enters latency, but can reactivate to cause herpes zoster in the same lumbar dermatome. The subsequent herpes zoster rash often covers a large portion of the dermatome outside of the area immediately surrounding the injection site. Based on the PRV round trip model for amplification of the virus in the submandibular gland, we propose a VZV model shown in Figure 1. In this model, VZV establishes latency in only a few neurons in the DRG after vaccination. When vaccine virus reactivates, progeny VZV enters axons and travels in the anterograde direction to the original injection site in the skin. Here it infects more skin cells and replicates, after which progeny virus travels on its first retrograde round trip in axons to the DRG, seeding more DRG neurons. Within the DRG, virus replicates in additional soma and progeny virions travel in axons in the anterograde direction from newly infected neurons in a second trip to the skin, where further virus replication expands the dermatomal distribution of the rash. Herpes virus particles travel at a rate of 10 to 12 cm per day in sensory neurons, while an infectious cycle is 10 to 16 hours (time required to produce new progeny virions).^10^ Therefore, at least two round trips between DRG and skin may be possible during a bout of herpes zoster before an adaptive immune response blocks further trips. Furthermore, the round trip model may explain more severe cases of herpes zoster in children who have had wild‐type varicella, especially if the varicella exanthem had been mild with few vesicles.

**Figure 1:**
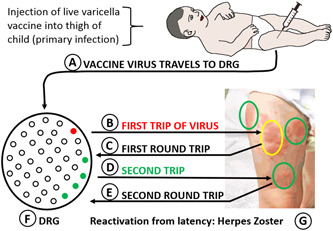
The round trip model for herpes zoster in immunized children based on pseudorabies neuronal experiments. Varicella immunization, usually in a thigh, leads to local viral replication, sometimes with transport of virus to the lumbar dorsal root ganglia (DRG), where the virus enters latency (pathway A). Months to years later, the vaccine virus can reactivate in the DRG and travel anterograde in sensory nerves to the site of the original infection in skin (pathway B; red arrow). The virus replicates locally and causes a rash (yellow circle). Thereafter, progeny virions engage more sensory nerve endings and are transported retrograde to the DRG in adjacent sensory fibers (pathway C). These virions in turn undergo a replication cycle and new progeny virions travel anterograde to the skin (pathway D; green arrow). Further local viral replication occurs at new sites in the skin (green circles) adjacent to the first site (yellow circle), further enlarging the dermatomal rash associated with herpes zoster. Progeny virions may travel retrograde on a second round trip (pathway E), until further viral replication is halted by the VZV adaptive immune response. The DRG is marked by letter F; the red circle in DRG indicates the neuron that was originally infected from the vaccine virus immunization (pathway A); the green neurons in DRG indicate neurons that were infected by virus that traveled retrograde from skin (round trips in pathways C and E) after the herpes zoster rash first appeared. The photo in panel G shows a zoster rash on the right thigh at the same location where child received his vaccination 1 year earlier (6). Since his rash was not typed, we also note similarity with a similar severe zoster rash in right thigh of another child with vaccine typing (see Figure 1, Reference 7). The latter rash was so severe that the child required acyclovir treatment

Based on published reports about the severe‐combined immunodeficient (SCID) mouse model for varicella, we think it highly unlikely that subclinical spread of vaccine virus in skin immediately after immunization can account for subsequent severe dermatomal herpes zoster. Studies in the SCID model have established that vaccine virus does not spread widely after injection into human skin xenografts.^11^ We have also found a change in distribution of herpes zoster in children at the University of Iowa medical center over the past 30 years.^12^ We tabulated the dermatomal distribution of herpes zoster following wild‐type varicella in our oncology population, before approval of varicella vaccination in 1995; we found that only 12% were lumbosacral. Although the number of childhood cases of herpes zoster has declined at our medical center since initiation of universal varicella vaccination, the percent that are lumbar has steadily increased over the past 2 decades. By 2014, we have observed that most cases of lumbar herpes zoster in children have occurred at the site of varicella vaccination (usually L2‐4 dermatomes).^6^ Even after the most severe rashes, however, we have not observed clinical signs of persistent ganglionitis, since all of our cases of vaccine‐related herpes zoster in immunocompetent children have had complete recovery of function in the affected dermatome without post‐herpetic neuralgia on follow‐up examinations. Epidemiology studies across the United States have confirmed that the incidence of herpes zoster in children is declining and probably will plateau at around 0.2 per 1000.^13^ Although the vaccine virus remains neurotropic and establishes latency similarly to wild‐type virus, vaccine virus does not reactivate as efficiently.^14^ For all the above reasons, universal varicella vaccination remains an effective method to prevent the most serious complications of wild‐type varicella in children.^15^
